# The complete mitochondrial genome of the vulnerable Australian crest-tailed mulgara (*Dasycercus cristicauda*)

**DOI:** 10.1080/23802359.2021.1911703

**Published:** 2021-04-22

**Authors:** Jaco D. Zandberg, Wayne G. Reeve, Frances Brigg, Serina M. McConnell, Peter B. S. Spencer

**Affiliations:** a Medical, Molecular and Forensic Sciences, Murdoch University, Murdoch, WA, Australia; b State Agricultural Biotechnology Centre, Murdoch University, Murdoch, WA, Australia; c Environmental and Conservation Sciences, Murdoch University, Murdoch, WA, Australia

**Keywords:** Chordata, crest-tailed Mulgara, D-loop, dasyuridae, marsupialia, mitogenome

## Abstract

In this announcement, we report the complete mitogenome of the vulnerable Crest-tailed Mulgara (*Dasycercus cristicauda*) (Krefft, 1867). The mitogenome was 17,085 bp in length and contained 13 protein-coding genes, two rRNA genes, 22 tRNAs and a 1583 bp variable control region (D-loop). The features of the *D. cristicauda* mitogenome are consistent with other vertebrate mitogenomes but, in contrast to other marsupials, appears to contain a functional tRNA-Lysine with a UUU anticodon. Phylogenetic analysis of available entire mitogenomes reveals it forms a cluster with other marsupials in the Dasyuromorphia order within the Australidelphian clade, being most closely related to the Northern Quoll and the Tasmanian Devil.

Mulgaras (*Dasycercus* sp.) are small 65–185 g, voracious, nocturnal marsupial predators that live in the deserts and spinifex grasslands of arid and semi-arid Australia feeding on smaller mammals and reptiles (Masters 2008). The identification of Mulgaras has been confusing and only recently has there been confidence in the taxonomic separation of the Mulgara genus *Dasycercus* into the two species *D. cristicauda* (the crest tailed Mulgara, previously *D. hillieri* and referred to as the Ampurta) and *Dasycercus blythi* (the Brush-tailed Mulgara, previously designated as *D. cristicauda*). Since there has been an decrease in range and numbers, the crest-tailed mulgara is currently listed as Vulnerable under the Australian Environment Protection and Biodiversity Conservation Act (EPBC Act, 1999). Although both species are very similar, they can be morphologically distinguished on the appearance of the tail (crested for *D. cristicauda* versus brushed for *D. blythi*), the number of upper premolars (three for *D. cristicauda* versus two for *D. blythi*) and the number of nipples (eight *D. cristicauda* versus six for *D. blythi;* Woolley 2005; Woolley et al. 2013). The inclusion of genetic data could enrich evolutionary studies of the Mulgaras taxon. In this study, we have, therefore, determined the complete mitogenome for *D. cristicauda* to enable genetic comparisons to be made to marsupials in the order Dasyuromorphia and to other vertebrates. The *D. cristicauda* DNA was sourced from voucher material from an individual located in the Simpson Desert Regional Reserve (−26.3225S; 136.6053E; lab number 17-065).

The total genomic DNA was sequenced using the Illumina MiSeq Platform (Illumina, San Diego, CA) to produce a total of 6,074,282 paired-reads reads (1.822 Gbp). The 300 bp paired-end reads were merged using BBMerge at a normal merge rate and subsequently trimmed by BBDuck at Q13. We then *de novo* assembled the complete mitogenome from 18,107 reads (0.94% of the total trimmed and merged reads) to produce a circular mitogenome of size 17,085 bp with 318× coverage. Annotations for the protein-coding, tRNA, and rRNA genes were retrieved by comparing the finished mitogenome to other finished marsupial mitogenomes using the ‘Annotate and Predict’ feature of Geneious Prime v2019.2.1 (Biomatters®, Auckland, New Zealand). The sequence with annotated features has been deposited in GenBank under the Accession number of MT762173. The *D. cristicauda* complete mitogenome has a typical vertebrate mitogenome organization (Nilsson et al. 2004; Westerman et al. 2016). It contained 13 protein-coding genes, two rRNA genes, 22 tRNA genes, and a non-coding control region (D-loop) of size 1583 bp. The overall base composition was 32.2% A, 30.6% T, 23.6% C, and 13.5% G, with a GC content of 37.1%. Twelve of the 13 protein-coding genes initiated with ATG, while one started with ATC (ND3). Eight protein-coding genes ended with TAA; four of which had TAA as the stop codon in the gene sequence (ND2, ND4L, ND5 and ND6) and the other four had the stop codon completed by the addition of 3′ A-residues to the mRNA (COX2, COX3, ND3 and ND4). Another four ended with TAG (ATP6, ATP8, COX1, ND1), and one ended with AGA (CYTB).

The mitogenome shares features consistent with other marsupial mitogenomes and contains the conserved tRNA “ACWNY” gene arrangement (Pääbo et al. 1991). Interestingly, *D. cristicauda* appears to have a functional tRNA-Lysine that contains the appropriate anticodon “UUU.” This anticodon is absent from the mitochondrial tRNA-Lysine of other marsupials and, hence, nuclear tRNA-Lysine is translocated into mitochondria containing the tRNA-Lysine pseudogene to restore function (Dörner et al. 2001). Direct comparison of all the curated reads used to assemble the Mulgara mitogenome to the closely related Tasmanian devil and Northern Quoll mitogenomes reveals that the tRNA-Lysine does indeed contain a functional “UUU” anticodon. Sequencing depth at anticodon position was 727 for the Tasmanian devil and 728 for the Northern Quoll.

Phylogenetic analysis was conducted using slowly evolving mitogenome sequences (excluding tRNA, intergenic regions and control regions) of sixteen finished mitogenomes (Figure 1), using the GTR + G + I model of best fit in MEGA-X (Nei and Kumar 2000). The phylogenetic comparison of revealed that *D. cristicauda* belongs to the Dasyuromorphia within the Australian Australidelphian clade (for which the name Euaustralidelphia “true Australidelphia” has been proposed) (Nilsson et al. 2010), with the closest relatives being the Northern Quoll and Tasmanian Devil.

**Figure 1.**
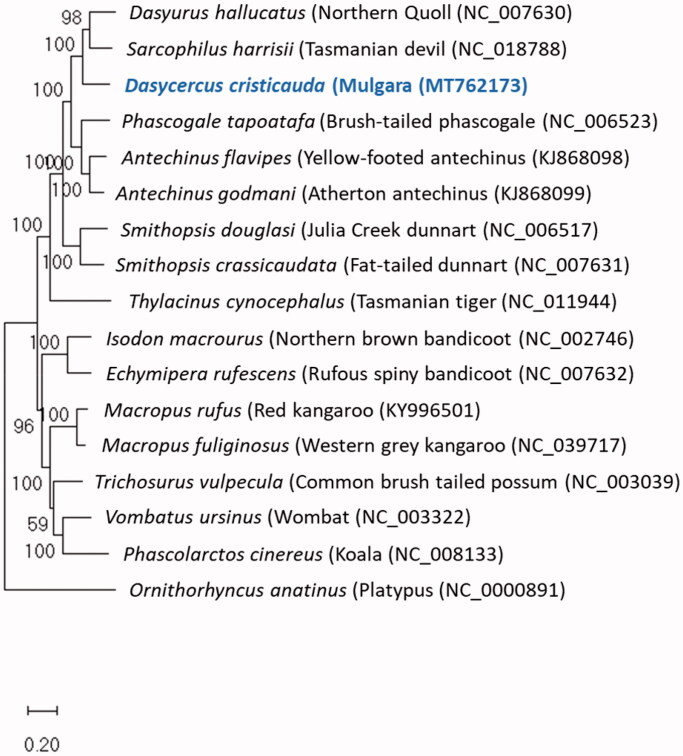
Phylogenetic placement of *Dasycercus cristicauda* based on a truncated comparison of the rRNA and coding DNA sequences to other entire vertebrate mitogenomes. The evolutionary history was inferred by using the Maximum Likelihood method and General Time Reversible model (Nei and Kumar 2000). The tree with the highest log likelihood (−257,475.18) is shown. The percentage of trees in which the associated taxa clustered together is shown next to the branches. Initial tree(s) for the heuristic search were obtained automatically by applying Neighbor-Join and BioNJ algorithms to a matrix of pairwise distances estimated using the maximum composite likelihood (MCL) approach, and then selecting the topology with superior log-likelihood value. A discrete Gamma distribution was used to model evolutionary rate differences among sites (five categories (+G, parameter = 0.6728)). The rate variation model allowed for some sites to be evolutionarily invariable ([+I], 19.05% sites). The tree is drawn to scale, with branch lengths measured in the number of substitutions per site. This analysis involved 28 nucleotide sequences. There were a total of 20348 positions in the final dataset. Evolutionary analyses were conducted in MEGA X (Kumar et al. 2018).

## Geolocation information

Geospatial coordinates from voucher material for the Crest-tailed Mulgara, *Dasycercus cristicauda*, originated from 50 km East of Purni Bore; Simpson Desert Regional Reserve, (-26.3225 S; 136.6053 E). S.A. Museum voucher: M19705/ABTC 37971.

## Data Availability

The data that support the findings of this study are available from either GenBank (see Figure 1) or from the corresponding author, [PS], upon reasonable request. The complete mitochondrial sequence has been deposited in the NCBI online database at https://www.ncbi.nlm.nih.gov/nuccore/MT762173.1/.
